# The Adrenal Gland Volume Measurements in Manifestation of the Metabolic Status in Type-2 Diabetes Mellitus Patients

**DOI:** 10.1155/2016/7195849

**Published:** 2016-08-02

**Authors:** Ismail Serifoglu, Ibrahim Ilker Oz, Muammer Bilici

**Affiliations:** ^1^Department of Radiology, Bagcilar Training and Research Hospital, Istanbul, Turkey; ^2^Department of Radiology, Bulent Ecevit University School of Medicine, Zonguldak, Turkey; ^3^Department of Internal Medicine, Bulent Ecevit University School of Medicine, Zonguldak, Turkey

## Abstract

*Objectives*. The aim of our study was to investigate the differences in adrenal gland volume between nondiabetic controls and Type-2 diabetic patients and to examine the influence of glycemic control in diabetes mellitus on adrenal gland volume.* Methods*. From March 2 to November 25, 2015, 62 consecutive patients with Type-2 DM along with 62 nondiabetics matched by age, gender, and BMI were enrolled in this prospective study. Our diabetes patients were categorized into two groups, well-controlled and poorly controlled diabetes groups. Adrenal volumetric measurements were performed by two radiologists, prospectively and independently, with semiautomatic software. Interobserver reliability was studied using the interobserver correlation coefficient (ICC).* Results*. The total adrenal volume (TAV) was significantly higher in Type-2 diabetic patients when compared with nondiabetic patients (*p* < 0.05). When we investigated diabetic patients according to glycemic controls, the TAVs in controlled diabetic patients were significantly higher than in those of the poorly controlled or uncontrolled diabetic patients (*p* < 0.05). Nondiabetic control patients have significantly smaller TAVs when compared to controlled and poorly or noncontrolled diabetic patients (*p* < 0.05).* Conclusion*. Our study suggests that adrenal gland volume measurement may be used as an indirect marker of glycemic control in patients with diabetes.

## 1. Introduction

Diabetes mellitus (DM) is a chronic disease causing impairment of the functions of many systems, such as the cardiovascular, immune, and central nervous systems. Its effect on the central nervous system is primarily caused by affecting the functions of the hypothalamic-pituitary-adrenal (HPA) axis.

The 24 h urine free cortisol (UFC) test is one of the best methods for the evaluation of the functions of the HPA axis. Alternatively, the collection of salivary cortisol and 1 mg overnight dexamethasone suppression test are also utilized [1]. It was reported that adrenal gland morphology is as effective as laboratory tests for the evaluation of HPA axis function [2]. The accuracy of the results of length measurement by means of multidetector computed tomography (MDCT) to assess the size of the adrenal glands is doubtful, since it is user-dependent and prone to alteration by section thickness. Alternatively, some recent studies have provided evidence that measuring adrenal volume by means of software programs using thin section MDCT images provides more reliable quantitative information [3, 4].

Studies investigating the association between diabetes and adrenal gland morphology have revealed that adrenal volume is increased in patients with diabetes [3, 5]. Hypoglycemia or hyperinsulinism in patients with DM stimulates the HPA axis. As a result of HPA activation, the adrenal cortex is stimulated for cortisol release [6, 7]. HPA hyperactivation and hypercortisolemia cause an increase in adrenal gland volume [8]. Animal studies have revealed that, in cases with uncontrolled or poorly controlled diabetes, persistent hyperglycemia impairs the functions of the HPA axis [9, 10]. It was shown that insulin treatment or endogenous insulin increase normalized the response of the HPA axis to hypoglycemia [11]. We are currently unaware of any study investigating the effect of glycemic control on adrenal gland size, which is a marker of the functions of HPA axis.

The aim of our study was to investigate the differences in adrenal volume between nondiabetic controls and Type-2 diabetic patients and to examine the influence of glycemic control in diabetes mellitus on adrenal gland volume.

## 2. Materials and Methods

### 2.1. Patients

From March 2 to November 25, 2015, all hospitalized Type-2 DM patients and nondiabetics admitted to the Endocrinology Department who had undergone abdominal computed tomography (CT) examination within three months were enrolled in this prospective study. Patients were diagnosed with Type-2 DM according to the American Diabetes Association (ADA) criteria [12]. We excluded those cases where they had any condition potentially affecting adrenal gland morphology (e.g., Cushing's syndrome, chronic steroid use, arterial hypertension, cancer, depression, or history of recent surgery) or if precise delineation of the adrenal gland volume from adjacent structures was not possible on CT images. Those patients with an adrenal nodule and known adrenal metastasis were also excluded. Finally, 62 consecutive patients with Type-2 DM along with 62 nondiabetics, matched by age, gender, and BMI, were enrolled (Table 1). These patients had Type-2 DM with a history of 10.31 ± 7.35 years and were treated by standard insulin therapy. This study protocol conforms to the ethical guidelines of the Declaration of Helsinki as reflected in a priori approval by our institution's human research committee. Informed written consent was obtained from all individuals.

### 2.2. Biochemical Analysis

A blood sample was withdrawn from all the participants after overnight fasting and plasma was separated by centrifugation at 3000 rpm for 10 minutes. Plasma glucose was assayed by an automated glucose oxidase method.

Glycemic control status of our diabetes patients was assessed by plasma A1C levels. A1C levels below 7%, which are the goal of the standard therapy, were accepted as representing controlled diabetes. Patients having A1C levels above 7% were classified as having poorly controlled or uncontrolled diabetes [12].

### 2.3. Computed Tomography Analysis

A multidetector CT system (Activion 16-row CT scanner; Toshiba Medical Systems, Otawara, Japan) was used for CT imaging. The routine abdominal CT protocols were used for all patients, these being 120 kV, 144 effective mAs, a pitch factor of 0.938, a helical factor of 15.0, a rotation time of 0.75 s, and a reconstruction interval of 0.5 mm. Patients were administered oral contrast medium (EZ-Cat, barium sulfate suspension; EZ-Em, Westbury, NY). The total of 100 mL nonionic contrast agent (Ultravist 370; Bayer Schering Pharma, Berlin, Germany) was given at a rate of 2.0 mL/s via peripheral venous line.

**Figure 1:**
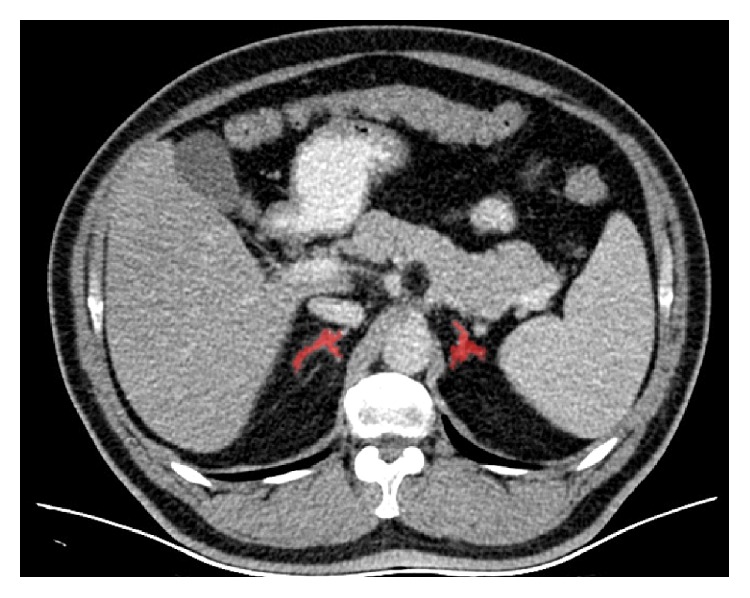
On abdominal contrast-enhanced MDCT image in axial plane was seen semiautomatically traced adrenal gland contour for each side.

**Figure 2:**
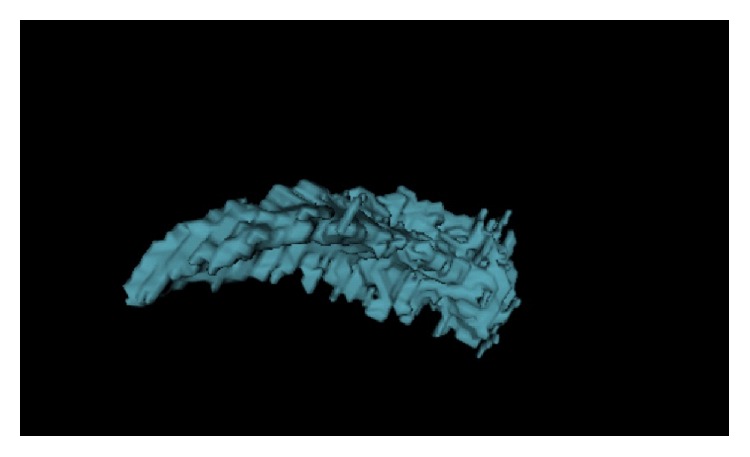
Three-dimensional reconstructed MDCT image shows posterolateral view of right adrenal gland.

An axial section thickness of 0.5 mm, a window level of 40 Hounsfield units (HU), and a width of 400 HU were used for image interpretation. The normal adrenal gland parenchyma was determined by 2-dimensional segmentation in the region. After tracing the adrenal gland contour semiautomatically for each side in all slices (Figure 1), the adrenal volume was automatically calculated by software (Figure 2). The total adrenal volume (TAV) was determined as the sum of the right and left adrenal gland volume measurements and is expressed in cm^3^.

For each patient, CT imaging analysis was measured by two independent radiologists with eight and six years of experience in abdomen imaging using image analysis software (OsiriX Foundation, Geneva, Switzerland) on a personal computer. The radiologists performing the measurements were “blinded” to the clinical information. The reviewers were able to manipulate the images to optimize the visualization of adrenal gland parenchyma. Intraclass correlation analysis shows the reproducibility estimate between two independent readers. The intraclass correlation coefficient (ICC) was excellent when measuring right, left, and total adrenal volume (0.983, 95% CI: 0.873–0.997; 0.974, 95% CI: 0.708–0.998; and 0.944, 95% CI: 0.708–0.995, resp.).

### 2.4. Statistical Analysis

Statistical analyses were performed using the R 3.2.3 software package. Descriptive statistics of continuous variables are given as the mean, standard deviation, median, minimum, and maximum values. Categorical variables are presented as frequencies and percentages. The Shapiro-Wilk test was used to test normality. Kruskal-Wallis test for three group comparisons and the Mann-Whitney *U* test was used for two group comparisons. To determine whether there are any significant relationships between the three or more independent groups we use one-way analysis of variance (ANOVA) test. Interrater reliability of the measurements of two independent observers was assessed with the intraclass correlation coefficient (ICC). Comparisons of categorical variables among groups were made using the Pearson Chi-square test. For all statistical comparisons, those with a *p* value below 0.05 were considered as statistically significant.

## 3. Results

Baseline characteristics of the study subjects are shown in Table 1. No statistically significant difference was found in age (*p* = 0.193), gender (*p* = 0.472), and BMI (*p* = 0.797) between patients with Type-2 DM and nondiabetic patients. The A1C and plasma fasting glucose levels in diabetic patients are shown in Table 2.

The mean adrenal volumes for right and left adrenal gland and for total adrenal volume (TAV) in healthy controls and diabetic patients are shown in Table 3.

To rule out a possible effect of BMI on adrenal volume, with use of ANOVA test we found that BMI have no statistically significant effect on adrenal volume (*p* = 0.377).

The TAV was significantly higher in Type-2 diabetic patients versus nondiabetic patients (*p* < 0.05) (Table 3). When we investigated diabetic patients according to glycemic controls, the TAVs in controlled diabetic patients were significantly higher than those of the poorly or uncontrolled diabetic patients (*p* < 0.05) (Table 4).

Nondiabetic control patients have significantly smaller TAV compared to controlled and poorly or noncontrolled diabetic patients (*p* < 0.05) (Table 4).

Intraclass correlation analysis shows the reproducibility estimate between two independent readers. Intraclass correlation coefficient (ICC) was excellent when measuring right and left adrenal gland volume (0.983, 95% CI: 0.972–0.990 and 0.974, 95% CI: 0.958–0.984, resp.), and total adrenal volume (0.988, 95% CI: 0.981–0.993).

## 4. Discussion

In agreement with previous reports, we found in our study that total adrenal volume (TAV) was significantly higher in Type-2 diabetic patients [3, 5]. This is linked to increased activity of the HPA axis in patients with diabetes. The stressors, such as hypoglycemia, induce the release of corticotropin-releasing hormone (CRH) from the paraventricular nucleus (PVN) of the hypothalamus. The CRH then stimulates the pituitary gland and increases synthesis of ACTH from proopiomelanocortin (POMC). Increased serum ACTH levels can cause hypercortisolism and adrenocortical growth. This hormonal alteration may be due to reduced relative feedback sensitivity to glucocorticoids in different parts of the axis, changes in 11-beta-hydroxysteroid dehydrogenase (11B-HSD) enzyme activity, and increased corticotropin-releasing hormone (CRH) [7, 13]. As observed in animal studies, an enlargement of adrenal glands belongs to the most typical signs of chronic stress and one of the most severe chronic stress states is considered to be untreated diabetes [14]. A study by Godoy-Matos et al. [5] found a significant increase in TAV of Type-2 diabetic patients compared to the nondiabetic control group. Similarly, in a multivariable study by Carsin-Vu et al. [3] investigating factors influencing adrenal gland volume, TAV was found to be significantly greater in diabetic subjects compared to the nondiabetic controls.

When the patients with Type-2 DM in our study were grouped as well-controlled and poorly controlled subjects, both groups had significantly increased TAV levels compared to the nondiabetic control subjects. This suggests that adrenal volume increases independently of the status of glycemic control in patients with Type-2 DM compared with the control patients. This may be explained by the irreversible persistence of changes in adrenal volume induced by the HPA axis activation caused by hypoglycemia and hyperinsulinism secondary to insulin resistance at the onset of Type-2 DM. Elahi-Moghaddam et al. [10] in a study on rats found that serum corticosterone levels were lower in subjects under hyperglycemic conditions than in the control group; hence the HPA axis functions were impaired. Inouye et al. [7] speculated that impaired epinephrine counterregulation in untreated diabetic rats blunted corticosterone response to hypoglycemia. In another study of Inouye et al. [15] it was stated that in uncontrolled diabetic rats the hypoglycemia was associated with impaired rise in CRH expression from the hypothalamus and lower corticosterone levels. Chan et al. [11] in study on rats observed that the response of the HPA axis was improved and cortisol levels increased after insulin was administered to diabetic subjects. Fruehwald-Schultes et al. [6] demonstrated that plasma ACTH levels were increased after high rate insulin infusion. In our study, patients with Type-2 diabetes were categorized into well-controlled and uncontrolled groups. In spite of the abovementioned facts [6, 7, 10, 11, 15] it is somewhat surprising that the well-controlled group showed significantly greater rather than smaller TAV. However, not only exaggerated but also inadequate HPA activation in response to demanding stimuli may have negative consequences. Consistently, patients with an anxiety disorder showed attenuated response to hypoglycemia compared to healthy controls [16].

The two primary techniques for evaluating the metabolic status in Type-2 diabetes mellitus patients are patient self-monitoring of blood glucose levels or interstitial glucose and A1C. Blood glucose levels monitoring is an instrument- and user-dependant method, whereas monitoring A1C levels is not reliable in children, teens, and young adolescent patients. In patients prone to glycemic variability, treatment judgment was given by the combination of results of A1C and blood glucose levels [12]. Our opinion is that evaluation of TAV in diabetic patients may be used as a supplemental tool for monitoring A1C and blood glucose levels in showing long-term metabolic status of diabetes mellitus.

Our study has several limitations. Firstly there are differences between groups in our study, but the values are overlapping. There are needs for further research in this field. Also we performed adrenal size evaluation by only measuring the adrenal volume on CT scan images. CT is considered to be the most accurate technique for evaluation of organ volume [17]. According to the results of the prior studies, adrenal volume measurement is more suitable for use by virtue of its reproducibility and high interobserver accuracy [4]. In line with the literature data, our study demonstrated a high interobserver accuracy for adrenal volume measurements. Our study group only included patients under standard insulin therapy. Prospective studies are needed in patients under intensive insulin therapy, which has being increasingly used to reduce diabetic complications.

## 5. Conclusions

We have demonstrated for the first time that there is a negative association between glycemic status and adrenal gland volume. We believe that the strict control of diabetes and long-term high-dose insulin therapy via HPA axis stimulation increase the TAV as an indirect indicator of the adrenal gland activation.

In conclusion, the present result can motivate scientist to further research to prove the suggestion that measurement of the adrenal gland volume may be used as an indirect marker of glycemic control in patients with diabetes.

## Figures and Tables

**Table 1:** Demographic characteristics of patients.

	Type-2 diabetes (*n* = 62)	Nondiabetes (*n* = 62)	*p* value
Males/females (%)	27/35 (46.6/53%)	31/31 (50%)	0.472^a^
Age (years)	60 (22–85)	57.5 (19–80)	0.093^b^
BMI	28.4 (19.9–53.7)	29.1 (19–41.7)	0.797^c^

^a^ *p* value was calculated using the Chi-square test.

^b^ *p* value was calculated using the *t*-test.

^c^ *p* value was calculated using the Mann-Whitney *U* test.

**Table 2:** A1C and plasma fasting glucose levels in diabetic patients.

	Controlled diabetes (*n* = 33)	Noncontrolled diabetes (*n* = 29)
Plasma fasting glucose (mg/dL)	147 (89–230)	220 (94–584)
A1C (%)	6.8 (5.8–7)	9.9 (7.2–13.2)

**Table 3:** Adrenal volumes in diabetic patients and control patients.

	Type-2 diabetes (*n* = 62)	Nondiabetes (*n* = 62)	*p* value^a^
Right adrenal volume (cm^3^)	2.42 (0.73–5.18)	1.83 (0.67–4.2)	0.000
Left adrenal volume (cm^3^)	3.10 (1.31–5.88)	2.24 (1.10–5.12)	0.000
Total adrenal volume (cm^3^)	5.53 (2.67–9.92)	4.03 (1.93–7.52)	0.000

^a^ *p* value was calculated using the Mann-Whitney test.

**Table 4:** Adrenal volumes in controlled and uncontrolled diabetic patients and control patients.

	Controlled diabetes (*n* = 33)	Noncontrolled diabetes (*n* = 29)	Nondiabetes (*n* = 62)	*p* value^a^
Right adrenal volume (cm^3^)	2.9 (1.21–5.18)	2.24 (0.73–4.16)	1.83 (0.67–4.2)	0.022
Left adrenal volume (cm^3^)	3.75 (2.03–5.88)	2.81 (1.31–4.7)	2.24 (1.10–5.12)	0.009
Total adrenal volume (cm^3^)	6.58 (3.93–9.92)	5.06 (2.67–8.46)	4.03 (1.93–7.52)	0.010

^a^ *p* value was calculated using the Kruskal-Wallis test.
